# Exploring the Role of Killer Cell Immunoglobulin-Like Receptors and Their HLA Class I Ligands in Autoimmune Hepatitis

**DOI:** 10.1371/journal.pone.0146086

**Published:** 2016-01-08

**Authors:** Roberto Littera, Luchino Chessa, Simona Onali, Francesco Figorilli, Sara Lai, Luca Secci, Giorgio La Nasa, Giovanni Caocci, Marcella Arras, Maurizio Melis, Sara Cappellini, Cinzia Balestrieri, Giancarlo Serra, Maria Conti, Teresa Zolfino, Michele Casale, Stefania Casu, Maria Cristina Pasetto, Lucia Barca, Claudia Salustro, Laura Matta, Rosetta Scioscia, Fausto Zamboni, Gavino Faa, Sandro Orrù, Carlo Carcassi

**Affiliations:** 1 Regional Transplant Center, R. Binaghi Hospital, ASL 8, Cagliari, Italy; 2 Center for the Study of Liver Diseases, Department of Medical Sciences "M. Aresu", University of Cagliari, Cagliari, Italy; 3 Department of Internal Medicine, University Hospital, Cagliari, Italy; 4 Medical Genetics, R. Binaghi Hospital, ASL 8, Cagliari, Italy; 5 Bone Marrow Transplant Center, R. Binaghi Hospital, ASL 8, Cagliari, Italy; 6 Hematology Unit, Department of Medical Sciences "M. Aresu", University of Cagliari, Cagliari, Italy; 7 Departmental Unit of Liver Transplantation, G. Brotzu Hospital, Cagliari, Italy; 8 Department of General Surgery, Division of liver and pancreas transplantation, G. Brotzu Hospital, Cagliari, Italy; 9 Department of Surgical Science, Division of Pathology, University of Cagliari, Cagliari, Italy; 10 Medical Genetics, Department of Medical Sciences "M. Aresu", University of Cagliari, Cagliari, Italy; University of Colorado Denver, UNITED STATES

## Abstract

**Background:**

Natural killer cells are involved in the complex mechanisms underlying autoimmune diseases but few studies have investigated their role in autoimmune hepatitis. Killer immunoglobulin-like receptors are key regulators of natural killer cell-mediated immune responses.

**Methods and Findings:**

KIR gene frequencies, KIR haplotypes, KIR ligands and combinations of KIRs and their HLA Class I ligands were investigated in 114 patients diagnosed with type 1 autoimmune hepatitis and compared with a group of 221 healthy controls. HLA Class I and Class II antigen frequencies were compared to those of 551 healthy unrelated families representative of the Sardinian population. In our cohort, type 1 autoimmune hepatitis was strongly associated with the HLA-B18, Cw5, DR3 haplotype. The *KIR2DS1* activating KIR gene and the high affinity HLA-C2 ligands were significantly higher in patients compared to controls. Patients also had a reduced frequency of HLA-Bw4 ligands for KIR3DL1 and HLA-C1 ligands for KIR2DL3. Age at onset was significantly associated with the *KIR2DS1* activating gene but not with HLA-C1 or HLA-C2 ligand groups.

**Conclusions:**

The activating KIR gene *KIR2DS1* resulted to have an important predictive potential for early onset of type 1 autoimmune hepatitis. Additionally, the low frequency of the KIR-ligand combinations KIR3DL1/HLA-Bw4 and KIR2DL3/HLA-C1 coupled to the high frequency of the HLA-C2 high affinity ligands for KIR2DS1 could contribute to unwanted NK cell autoreactivity in AIH-1.

## Introduction

Autoimmune hepatitis (AIH) is a rare chronic liver disorder [1] characterized by loss of immunological tolerance to autologous liver tissue. This breakage in the immune system typically causes hepatocellular inflammation with high levels of circulating autoantibodies, hypergammaglobulinemia and fluctuating increases of serum transaminases and immunoglobulin G levels. Along with primary biliary cirrhosis (PBC) and primary sclerosing cholangitis (PSC), AIH represents one of three main categories of autoimmune liver disease. Although more than 40% of patients have an acute onset, the disease may be asymptomatic, and in some cases present as fulminant hepatitis [2, 3, 4].

The diagnosis of AIH is primarily based on the scoring system established by the International Autoimmune Hepatitis Group (IAIHG) in 1993 and revised in 1999 [5]. A simplified scoring system was introduced in 2008 for wider applicability in clinical practice [6]. Updated diagnostic and treatment strategies are published in the guidelines of the American Association for the Study of Liver Diseases (AASLD).

Histological features of AIH include periportal hepatitis with lymphocytic infiltrates, plasma cells, and piecemeal necrosis but there is no specific histological feature capable of confirming the diagnosis of AIH. To formulate the diagnosis, chronic liver conditions such as viral hepatitis, drug- or alcohol-induced hepatitis, fatty liver disease and metabolic disorders need to be excluded [7, 8].

Circulating autoantibodies are a distinguishing feature of AIH. Since the discovery of autoantibodies directed against different cellular targets including endoplasmatic reticulum membrane proteins, nuclear antigens and cytosolic antigens, AIH has been classified according to serum autoantibody profiles.

Type I AIH (AIH-1) is the most common subclass (80%) and is mainly characterized by circulating antinuclear antibodies (ANA) and/or smooth muscle antibodies (SMA), as well as soluble liver antigen (SLA) antibodies or antibodies against the liver pancreas antigen (LP), designated together as SLA/LP.

Type II AIH is characterized by anti-liver/kidney microsomal antibody type 1 (anti-LKM1) or anti-LKM type 3 (anti-LKM3) and/or antibodies against anti-liver cytosol type 1 antigen (anti-LC1) [9, 10, 11].

Persistent liver inflammation resulting from an overly aggressive autoimmune reaction against hepatocytes is the hallmark of AIH. Although the mechanisms underlying loss of self-tolerance remain speculative, impaired negative selection of autoreactive lymphocytes [12–14] and the clonal expansion of immunocytes cross-reactive to homologous antigens (molecular mimicry) [15–17] are likely to be involved.

Within this context, it is understandable that certain antigens, alleles or allelic groups of the major histocompatibility complex (MHC) confer susceptibility to AIH. Particularly the human leukocyte antigen (HLA) class II alleles DRB1*03:01 and DRB1*04:01 that encode a lysine at position 71 of the antigen-binding groove would seem to permit binding with liver specific self-antigens, thereby leading to activation of CD4^+^ and CD8^+^ T cells [18, 19]. It has been suggested that also HLA Class I alleles in strong linkage disequilibrium with DRB1*03:01 and DRB1*04:01 may contribute to disease susceptibility [20–22]. In the North American population, it has been shown that the HLA-A1, B8, DR3 haplotype is an independent risk factor for AIH [23]. Another report comparing Italian to North American AIH patients found a significant association for the HLA-B8, Cw7, DR3, DQ2 haplotype [21].

One of the potential immunopathogenetic pathways implicated in AIH is the reduced number and function of CD4^+^ and CD25^+^ regulatory T-cells (Tregs). It is widely recognized that these cells help control cytokine production mediated by CD4^+^ helper T cells and CD8^+^ cytotoxic T lymphocytes (CTL) and limit the function of macrophages, dendritic cells, B cells and natural killer (NK) cells [12, 15].

Although numerous scientific reports have highlighted the involvement of CD4^+^ and CD8^+^ T cells in the immunopathogenetic mechanisms of AIH, there are few reports investigating the role of NK cells. NK cells (CD16^+^ and CD56^+^) are a heterogeneous subpopulation of lymphocytes that have a central role in innate immunity and strongly contribute to the regulation of adaptive immune responses [24].

NK cell function is governed by the delicate interplay between positive and negative signaling receptors that ultimately determines NK cell reactivity after encounters with target cells. Killer immunoglobulin-like receptors (KIRs), expressed on the NK cell surface, have a key role among the regulators of NK cell activity.

KIR genes have been divided into two broad groups of haplotypes according to the following criteria. Haplotypes pertaining to Group A contain a fixed number of genes (the *KIR3DL3*, *KIR2DL1*, *KIR2DL3*, *KIR2DL4*, *KIR3DL1*, and *KIR3DL2* inhibitory KIR genes and the *KIR2DS4* activating KIR gene) whereas those of Group B contain different combinations of both activating and inhibitory KIR genes including 1 to 8 of the specific genes, *KIR2DS1*, *KIR2DS2*, *KIR2DS3*, *KIR2DS5*, *KIR3DS1*, *KIR2DL2*, KIR2DL5A and *KIR2DL5B* [25, 26].

The function of KIR receptors depends strongly upon their interaction with HLA class I ligands.

HLA-C is the predominant KIR ligand. Interaction between KIR and HLA-C is influenced by a single amino acid substitution at position 80 of the alpha-1 domain of the alpha helix. HLA-C group 1 molecules (C1) have an asparagine residue at this position whereas HLA-C group 2 molecules (C2) have a lysine. [27, 28].

The KIR2DL2 and KIR2DL3 inhibitory receptors and the KIR2DS2 activating receptor recognize molecules of the C1 group while the KIR2DL1 inhibitory receptor and the KIR2DS1 activating receptor recognize molecules of the C2 group. The KIR3DL1 inhibitory receptor and, possibly, the KIR3DS1 activating receptor bind HLA-B molecules with the serologically defined epitope Bw4. Howeverfurther complications arise from variations in KIR-ligand interactions and binding affinities. For example, it has been shown that some alleles of *KIR2DL2* and *KIR2DL3* interact with specific HLA antigens of the C2 group [29].

NK cells only represent 5–10% of peripheral blood circulating lymphocytes but in liver tissue constitute about 30% of the total lymphocytic population, reaching 50% in liver diseases [30] where they have an important role in controlling hepatotropic viral replication and protecting the host against the development of hepatocellular carcinoma (HCC) related to hepatitis B (HBV) and C (HCV) viruses [31, 32].

In view of these considerations, it is plausible to hypothesize that NK cells are also involved in the pathogenesis of autoimmune liver disease and in particular AIH.

The present study investigated the impact of NK cells on the development and course of the disease with particular emphasis on KIR gene repertoire and the role of HLA class I molecules in their dual function as high-affinity ligands for KIR receptors and activators of CD8^+^ T cells.

## Materials and Methods

The cohort comprised 114 Sardinian outpatients with type I AIH, median age 55 years [interquartile range (IQR) 41–68 years], who referred to the Center for the Study of Liver Diseases, Department of Medical Sciences "M. Aresu", University of Cagliari, Italy. The diagnosis of all patients was based on the IAIHG revised scoring system and AASLD guidelines [6]. Patients with type II AIH or overlap syndrome were excluded as well as patients with other chronic liver diseases such as drug- or alcohol-induced hepatitis, fatty liver disease, metabolic disorders, genetic disorders, hereditary conditions such as Wilson disease, autoimmune cholangitis, primary biliary cirrhosis and primary sclerosing cholangitis.

All enrolled patients were seronegative for HBsAg and for anti-hepatitis A virus IgM antibody, anti-hepatitis C virus IgG antibody, anti-hepatitis D virus IgG antibody, and anti-hepatitis E virus IgM antibody.

The HLA class I and class II antigen frequencies (HLA-A, B, C, DR) observed in our patients were compared to those of a study carried out on 551 healthy unrelated families representative of the Sardinian population [33]. HLA haplotypes in AIH-1 patients were obtained by the maximum likelihood method using the Arlequin computer program, version 3.0 [34].

KIRs and their HLA Class I ligands were compared between 114 AIH-1 patients and 221 healthy controls enrolled in the Sardinian Voluntary Bone Marrow Donor Registry.

### Ethics Statement

Patients were recruited and enrolled in the study protocol at the Center for the Study of Liver Diseases, Department of Medical Sciences "M. Aresu", University of Cagliari and the Departmental Unit of Liver Transplantation of the G. Brotzu Hospital in Cagliari, Italy.

Written informed consent was obtained from each patient included in the study in accordance with the ethical standards (institutional and national) of the local human research committee. The study protocol, including informed consent procedures, conforms to the ethical guidelines of the Declaration of Helsinki and was approved by the responsible ethics committee (Ethics Committee of the Cagliari University Hospital; date of approval: January, 23, 2014; protocol number NP/2014/456). Records of written informed consent are kept on file and are included in the clinical record of each patient.

### HLA and KIR genotyping

HLA and KIR genotyping was performed on genomic DNA extracted from peripheral blood mononuclear cells according to standard methods. Patients and controls were typed at high resolution for the alleles at the HLA-A, -B, -C and DR loci using a polymerase chain reaction sequence-specific primer (PCR-SSP) method according to the manufacturer’s instructions (Allele-specific PCR-SSP kits: Olerup SSP AB, Stockholm, Sweden). When comparing our data with those of previous reports we refer to alleles, allelic groups or serological equivalents, as appropriate.

HLA-C antigens were assigned to the C1 or C2 ligand group according to the presence of asparagine or lysine at position 80 of the HLA-C molecule [27, 28].

HLA-B antigens were classified as either Bw4 or Bw6 according to the amino acid positions spanning positions 77–83. HLA-A23, -A24, and -A32 pertain to the HLA-Bw4 group of serological epitopes.

Genomic DNA from both patients and controls was typed for the presence of the 14 KIR genes *KIR2DL1*, *KIR2DL2*, *KIR2DL3*, *KIR2DL4*, *KIR2DL5*, *KIR3DL1*, *KIR3DL2*, *KIR3DL3*, *KIR2DS1*, *KIR2DS2*, *KIR2DS3*, *KIR2DS4*, *KIR2DS5* and *KIR3DS1* using PCR with primers specific for each locus according to a previously reported method [35, 36].

### Haplotype group assignment and KIR-ligand combinations

KIR B haplotypes are characterized by the presence of one or more of the following genes: *KIR2DL5*, *KIR2DS1*, *KIR2DS2*, *KIR2DS3*, *KIR2DS5* and *KIR3DS1*. Conversely, these genes are not present on KIR A haplotypes [37]. Based on the presence or absence of these genes, patients homozygous for KIR haplotype A (KIR genotype AA) could be distinguished from patients heterozygous or homozygous for KIR haplotype B (KIR genotypes AB or BB, referred together as KIR genotype Bx) [38].

Particular attention was dedicated to KIR-ligand combinations because of the essential role of their interactions in the educational process of functional and potentially alloreactive NK cell clones.

### Statistical analysis

Summary statistics for clinical and biochemical parameters of patients with AIH-1 are presented in Table 1: medians, IQR, mean and standard deviations (SD) were calculated for all continuous variables; percentages and 95% confidence intervals (95% CI) were computed for categorical data.

**Table 1:** Baseline clinical and biochemical parameters of patients affected by type 1 autoimmune hepatitis.

Number of patients	114	
Gender: n (%)	F: 100 (87.7) M: 14 (12.3)	
	**median (IQR)**	**mean ± SD**
**Age (years)**	55 (41–68)	54 ± 17
**Age at diagnosis (years)**	49 (37–61)	47 ± 18
**AST level (IU/L)**	21 (18–30)	59 ± 171
**ALT level (IU/L)**	22 (15–29)	64 ± 196
**ALP level (IU/L)**	99 (56–170)	115 ± 73
**Bilirubin level (μmol/L)**	11.1 (10.3–15.1)	14.2 ± 8.4
**Albumin level (g/dL)**	4.0 (3.8–4.2)	3.9 ± 0.4
**γ-globulin level (g/dL)**	1.4 (1.1–1.6)	1.4 ± 0.6
**PT-INR**	1.00 (1.00–1.04)	1.02 ± 0.06
	**Number of patients (%)**	**95% CI**
**ANA positivity**	74 (64.9)	56.2–73.7
**SMA positivity**	48 (42.1)	33.0–51.2
**LKM1/LKM3 positivity**	0	
**AMA positivity**	0	
**Autoimmune diseases**	44 (38.6)	29.7–47.5
**Hepatitis B and C**	0	
**HLA-DR3 antigen**	64 (56.1)	47.0–65.2
**HLA-DR4 antigen**	34 (29.8)	21.4–38.2
**Prednisone therapy**	80 (70.2)	61.8–78.6
**Azathioprine therapy**	66 (57.9)	48.8–67.0
**UDCA therapy**	16 (14.0)	7.7–20.4
**Poor response to therapy**	6 (5.3)	1.2–9.4

To compare the HLA antigen and haplotype frequencies of AIH-1 patients with those of the Sardinian population we used the chi-squared test with Bonferroni correction. For each HLA haplotype the corrected P value (P_c_) was calculated by multiplying the P value obtained with the chi-squared test by the number of possible antigenic combinations. For each single HLA antigen, P_c_ was obtained by multiplying the uncorrected P value by the number of antigens observed. Only P_c_ values lower than 0.05 were considered to be statistically significant.

For two-locus haplotypes the expected frequency was calculated as the product of the frequencies of the component antigens, as proposed by Lewontin [39] and discussed by Contu et al. [33]. For three- and four-locus haplotypes the expected frequency was computed according to the formulas proposed by Thomson and Baur [40] and Slatkin [41], respectively. Linkage Disequilibrium (LD) was measured by the parameter *D*, given by the difference between the observed and expected frequencies. *D′* is the parameter *D* normalised to one (-1 ≤ *D′* ≤ 1). *D′* was computed as described by Robinson et al. [42].

Comparisons were made between patients and controls for KIR gene frequencies, KIR haplotypes, KIR ligands and combinations of KIRs and their HLA Class I ligands. Some of the observed and/or expected frequencies yielded values below 5 and so the chi-squared test could not be applied. Hence, we used the exact Fisher’s test to compute all P values and calculated the odds ratios (OR) with a 95% confidence interval (95% CI). The level of significance was set at 0.05.

**Fig 1:**
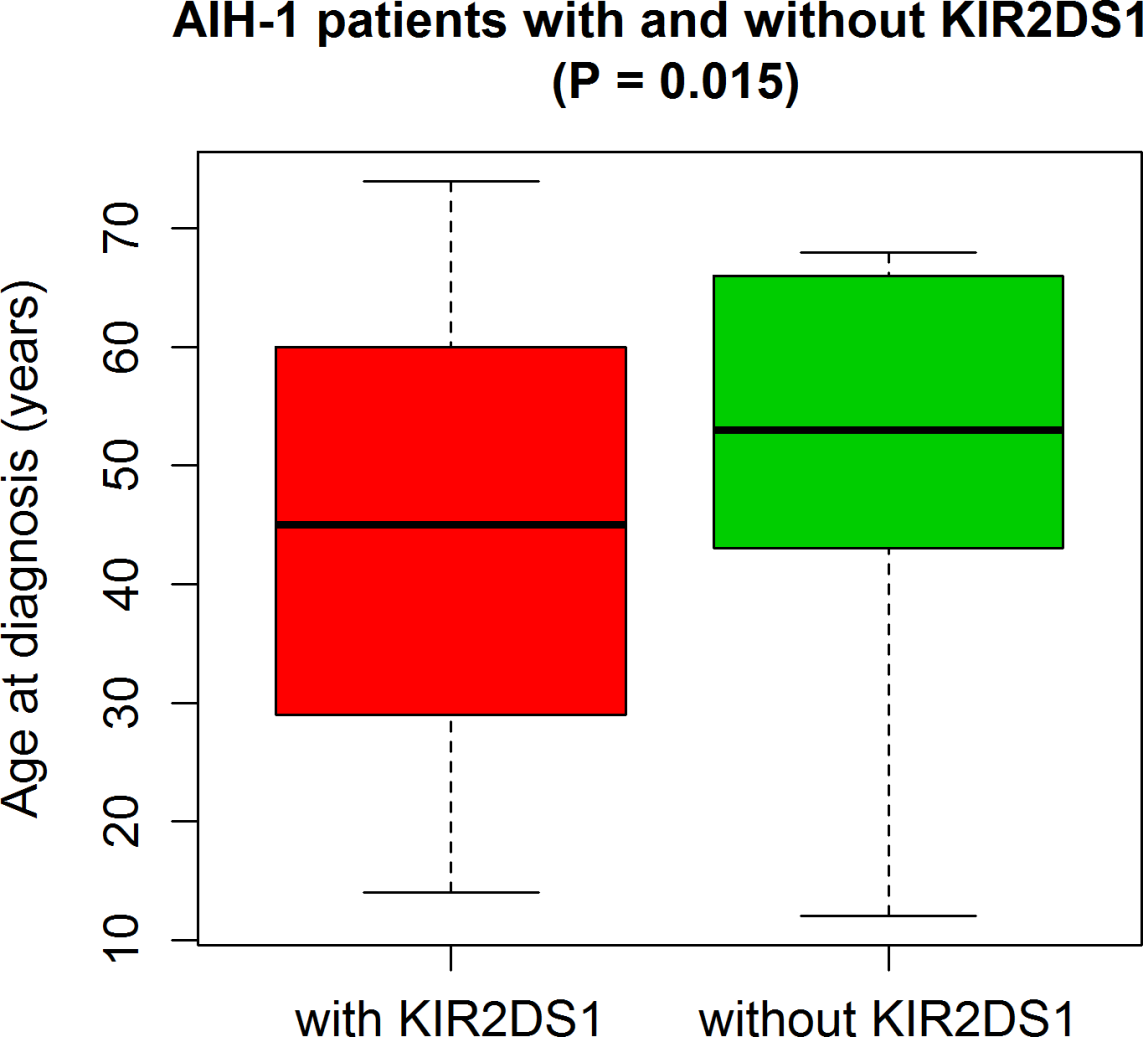
Predictive potential of the *KIR2DS1* activating KIR gene for early onset of AIH-1.

Because the difference between age at diagnosis in patients with and without *KIR2DS1* did not follow a normal distribution (as indicated by the Shapiro-Wilk test, the normal Q-Q plots and histograms of the data compared to the normal distribution plot), we used the non-parametric Mann-Whitney U test to compare these two groups (Fig 1).

Statistical analysis was performed using R version 3.2.0 The R Foundation for Statistical Computing, Vienna Austria).

## Results

Baseline and clinical characteristics of patients are reported in Table 1.

The median age at diagnosis was 49 years (IQR 37–61) and 100 patients (87.7%) were females. The median duration of disease was 6 years (IQR 2–10 years). Most patients had high titers of ANA (64.9%), occurring either alone or in association with SMA (42.1%). Anti-LKM-1 and anti-LKM-3 autoantibodies were absent in all study subjects. Nine patients (7.9%) tested negative for all AIH-related autoantibodies but were retained for analysis because of their high diagnostic scores.

A large percentage of patients (38.6%) presented one or more associated autoimmune disorders such as Hashimoto’s thyroiditis, rheumatoid arthritis, mixed connective tissue disease etc. About 75.4% of our patients typed positive for HLA-DR3 and/or HLA-DR4 antigens. The majority of patients (57.9%) had been treated with azathioprine, either alone (10.5%) or in combination with steroids (47.4%), and only 5.3% were non responders or partial responders.

The clinical and immunogenetic parameters of the Sardinian AIH-1 patients showed considerable overlap with those observed in AIH-1 patients of mainland Italy. Similar results were found in North American patients [21].

Also in our patient cohort, the susceptibility antigen HLA-DR3 had the highest frequency, yielding a statistically significant difference when compared to that observed by Contu et al. [33] for the Sardinian population (37.7% vs 25.7%, P_c_ = 1.4 x 10^−3^). In Sardinia, HLA-DR3 is generally inherited with the HLA-A30, B18, Cw5, DR3 haplotype. The frequency of this haplotype in our patients reached 21.1% compared to 14.6% of the general Sardinian population (P = 0.013). Additional analysis showed that the frequency of the HLA-B18, Cw5, DR3 three-loci haplotype was nearly twice as high in patients compared to controls (30.7% vs 17.8%, P_c_ = 1.4 x 10^−5^) (Table 2).

**Table 2:** Comparison of antigen and haplotype frequencies of AIH-1 patients with those of the Sardinian population.

	Sardinian Population (551 families, 2202 antigens)				AIH-1 cases (114 patients, 228 antigens)				Sardinian populationvsAIH-1 patients	
	Observedn (%)	Expectedn (%)	*D* (%)	*D′*	Observedn (%)	Expectedn (%)	*D* (%)	*D′*	*P value*	*Pc value*
**Complete HLA haplotype**										
HLA A30, Cw5, B18, DR3	322 (14.62)	223 (10.13)	4.51	1.00	48 (21.05)	40 (17.46)	3.59	1.00	0.013	**0.013**
**Partial HLA haplotypes**										
HLA A30, Cw5, B18	346 (15.71)	225 (10.23)	5.48	0.78	48 (21.05)	39 (17.08)	3.97	1.00	0.047	0.187
HLA Cw5, B18, DR3	392 (17.80)	261 (11.84)	5.96	0.65	70 (30.70)	57 (24.94)	5.76	1.00	3.5·10^−6^	**1.4·10^−5^**
HLA A30, B18, DR3	335 (15.21)	223 (10.12)	5.09	0.86	48 (21.05)	39 (17.31)	3.74	1.00	0.027	0.109
HLA A30, Cw5, DR3	322 (14.62)	208 (9.43)	5.19	0.69	48 (21.05)	38 (16.46)	4.59	1.00	0.013	0.053
HLA A30, Cw5	358 (16.25)	118 (5.35)	10.90	0.62	48 (21.05)	20 (8.59)	12.46	0.67	0.079	0.476
HLA A30, B18	374 (16.98)	137 (6.21)	10.77	0.64	50 (21.93)	25 (10.97)	10.96	0.68	0.075	0.449
HLA A30, DR3	352 (15.98)	130 (5.91)	10.07	0.59	48 (21.05)	23 (10.26)	10.79	0.64	0.061	0.369
HLA Cw5, B18	458 (20.79)	138 (6.27)	14.52	0.86	72 (31.58)	29 (12.74)	18.84	1.00	2.4·10^−4^	**1.5·10^−3^**
HLA Cw5, DR3	411 (18.66)	131 (5.97)	12.69	0.73	70 (30.70)	27 (11.91)	18.79	0.96	2.1·10^−5^	**1.3·10^−4^**
HLA B18, DR3	444 (20.16)	152 (6.92)	13.24	0.71	74 (32.46)	35 (15.22)	17.24	0.77	2.3·10^−5^	**1.4·10^−4^**
**Single HLA antigens**										
HLA-A30	507 (23.02)				62 (27.19)				0.183	1
HLA-Cw5	512 (23.25)				72 (31.58)				0.007	0.077
HLA-B18	594 (26.97)				92 (40.35)				2.7·10^−5^	**5.7·10^−4^**
HLA-DR3	565 (25.66)				86 (37.72)				1.3·10^−4^	**1.4·10^−3^**

Linkage Disequilibrium (LD) was measured by the parameter *D* normalised to one (*D′*). P values were calculated by the chi-squared test and corrected using Bonferroni’s method (P_c_).

Table 3 shows the results of comparisons between 114 AIH-1 patients and 221 healthy controls for the frequencies of activating and inhibitory KIR genes and KIR haplotypes.

**Table 3:** Comparisons of KIR gene frequencies and KIR haplotypes between patients with type 1 autoimmune hepatitis and controls.

	221 controlsn (%)	114 AIH-1 patientsn (%)	P value	OR (95% CI)
***Inhibitory KIR***				
**2DL1**	215 (97.3)	114 (100)	0.099	
**2DL2**	126 (57.0)	68 (59.6)	0.726	1.11 (0.69–1.81)
**2DL3**	188 (85.1)	104 (91.2)	0.123	1.82 (0.84–4.32)
**2DL4**	221 (100)	114 (100)	1	
**2DL5**	116 (52.5)	62 (54.4)	0.817	1.08 (0.67–1.74)
**3DL1**	206 (93.2)	108 (94.7)	0.644	1.31 (0.46–4.24)
**3DL2**	219 (99.1)	114 (100)	0.550	
**3DL3**	221 (100)	114 (100)	1	
***Activating KIR***				
**2DS1**	97 (43.9)	65 (57.0)	**0.028**	**1.69 (1.05–2.75)**
**2DS2**	125 (56.6)	64 (56.1)	1	0.98 (0.61–1.59)
**2DS3**	77 (34.8)	38 (33.3)	0.809	0.94 (0.56–1.55)
**2DS4**	202 (91.4)	110 (96.5)	0.109	2.58 (0.83–10.69)
**2DS5**	75 (33.9)	36 (31.6)	0.714	0.90 (0.54–1.49)
**3DS1**	88 (39.8)	46 (40.4)	1	1.02 (0.63–1.66)
***KIR Haplotypes***				
**AA**	66 (29.9)	28 (24.6)	0.369	0.77 (0.44–1.31)
**Bx**	155 (70.1)	86 (75.4)	0.369	1.31 (0.76–2.28)

Except for the *KIR2DS1* activating KIR gene, which had a significantly higher frequency in patients [57.0% vs 43.9%, OR 1.69 (95% CI 1.05–2.75), P = 0.028], no significant differences were observed between the two groups. Interestingly, patients carrying *KIR2DS1* also had a lower age at diagnosis compared to patients lacking this gene [median (IQR): 45 years (30–59) vs 53 years (44–66), P = 0.015].

The boxplots in Fig 1 illustrate the important predictive potential of the *KIR2DS1* activating KIR gene for early onset of AIH-1 (Fig 1).

Homozygosity for KIR A haplotype had a similar distribution within the two groups and could therefore be excluded as having a potential role in the development of disease.

Because HLA-Cw5 molecules belong to KIR ligand group C2 and HLA-B18 molecules are classified as Bw6, AIH patients carrying the HLA-B18, Cw5, DR3 three-loci haplotype will obviously display marked differences with respect to the control population for the C1, C2, Bw4 and Bw6 ligand groups. In fact, homozygosis for the C2 ligand group (C2/C2) was significantly higher in patients than in controls [43.9% vs 27.1%, OR 2.09 (95% CI 1.27–3.46), P = 0.003].

Consequently, patients also had a higher frequency of the KIR-ligand combination KIR2DS1/HLA-C2 present compared to the controls [48.2% vs 36.2%, OR 1.64 (95% CI 1.01–2.67), P = 0.035]. No significant differences were found between patients and controls for the other KIR-HLA ligand combinations.

Conversely, the frequency of epitopes pertaining to the HLA-Bw4 group was significantly reduced in patients [59.6% vs 79.2%, OR 0.39 (95% CI 0.23–0.66), P = 0.0003] (Table 4). This overexpression of HLA-C2 KIR ligands and the low frequency of HLA-Bw4 ligands explains the significantly reduced presence of the KIR2DL3/HLA-C1 and KIR3DL1/HLA-Bw4 KIR-ligand combinations in patients compared to controls [48.2% vs 61.1%, OR 0.59 (95% CI 0.37–0.96), P = 0.027 and 56.1% vs 71.9%, OR 0.50 (95% CI 0.30–0.82), P = 0.005, respectively] (Table 4).

**Table 4:** Comparisons of KIR ligands and combinations of KIR-HLA ligands between patients with type 1 autoimmune hepatitis and controls.

	221 controls n (%)	114 AIH-1 patients n (%)	P value	OR (95% CI)
***KIR Ligands*:**				
C1/C1	53 (24.0)	18 (15.8)	0.091	0.60 (0.31–1.10)
C2/C2	60 (27.1)	50 (43.9)	**0.003**	**2.09 (1.27–3.46)**
C1/C2	108 (48.9)	46 (40.4)	0.165	0.71 (0.44–1.15)
HLA-Bw4 present	175 (79.2)	68 (59.6)	**0.0003**	**0.39 (0.23–0.66)**
HLA-Bw6 present	206 (93.2)	106 (93.0)	1	0.96 (0.37–2.72)
***Activating KIR /Ligand*:**				
2DS1 /HLA-C2 present	80 (36.2)	55 (48.2)	**0.035**	**1.64 (1.01–2.67)**
2DS1 /HLA-C1/C1	20 (9.0)	10 (8.8)	1	0.97 (0.39–2.26)
2DS1 /HLA-C2/C2	42 (19.0)	26 (22.8)	0.474	1.26 (0.69–2.26)
2DS1 /HLA-C1/C2	38 (17.2)	29 (25.4)	0.084	1.64 (0.92–2.94)
2DS2 /HLA-C1 present	75 (33.9)	38 (33.3)	1	0.97 (0.58–1.61)
3DS1 /HLA-Bw4 present	66 (29.9)	24 (21.1)	0.092	0.63 (0.35–1.10)
***Inhibitory KIR /Ligand*:**				
2DL1 /HLA-C2 present	163 (73.8)	94 (84.2)	0.078	1.67 (0.92–3.12)
2DL2 /HLA-C1 present	78 (35.3)	38 (33.3)	0.809	0.92 (0.55–1.51)
2DL3 /HLA-C1 present	135 (61.1)	55 (48.2)	**0.027**	**0.59 (0.37–0.96)**
3DL1 /HLA-Bw4 present	159 (71.9)	64 (56.1)	**0.005**	**0.50 (0.30–0.82)**

Age at onset was significantly associated with the *KIR2DS1* activating gene but not with KIR C1 or C2 ligand groups, whether taken alone or considered in combination with *KIR2DS1* or other KIR genes. However, diagnosis was slightly delayed when *KIR2DS1*-positive patients were homozygous for HLA-C2/C2 compared to patients with HLA-C1/C2 or HLA-C1/C1 [median age at diagnosis (IQR): 53 years (25–63) vs 41 years (34–48) and 49 years (34–60), respectively; P not significant].

## Discussion

It has long been established that in Caucasoids AIH is associated with inheritance of the HLA-DR3 antigen. Indeed, in our study, this antigen had a higher frequency in patients compared to the general Sardinian population (37.7% vs 25.7%, P_c_ = 1.4 x 10^−3^) and thus confirms association of HLA-DR3 with AIH-1 also in the Sardinian population. However, a stronger association was found for the HLA-B18, Cw5, DR3 three-loci haplotype (Pc = 1.4 x 10^−5^). All patients with this haplotype presented the HLA-B*18:01, C*05:01, DRB1*03:01 alleles. This finding is different from that observed for mainland Italian and North American patients where AIH-1 is associated with the HLA-B8, DR3, DQ2 haplotype [21, 23].

Evidence continues to emerge that greater or lesser susceptibility to AIH-1 is not confined to HLA alleles of class II (HLA-DRB1*03, *04 and *13 confer greater susceptibility and HLA-DQB1*03 has a protective effect) [43–46] but also involves specific HLA alleles of class I [21, 47]. Particularly interesting within this context is the statistically significant higher frequency observed for the HLA-B*18:01 allele with respect to the HLA-DRB1*03:01 allele in the Sardinian AIH-1 patients (P_c_ = 5.7 x 10^−4^ vs P_c_ = 1.4 x 10^−3^). It is possible that HLA-B*18:01 coded molecules interfere with the ability of CD8^+^ lymphocytes to discriminate between self and nonself and thus promote the development and/or progression of the disease. An analogous mechanism has been proposed for other HLA molecules [48, 49].

The high frequency observed in our study for the HLA-B*18:01 and C*05:01 alleles translates into a low frequency of HLA-Bw4 KIR ligands and an overexpression of group C2 KIR ligands. This finding contrasts with the low frequencies of the HLA-C2 ligand group observed in North Italian PBC patients [49]. The different epitopes that characterize molecules of the C1 and C2 groups probably drive immune responses in the direction of the different cell targets that distinguish AIH from PBC.

The *KIR2DS1* activating gene is one of the best known and studied KIR genes and has often been indicated as a likely protagonist in human immune response mechanisms. High frequencies of this gene have also been described in many autoimmune disorders. In patients affected by systemic lupus erythematosus or scleroderma, *KIR2DS1* would seem to have a role in determining susceptibility [50]. Also in patients with psoriatic arthritis this gene has been found with a higher frequency compared to healthy controls and psoriatic patients without articular manifestations [51, 52]. In our study *KIR2DS1* had a significantly higher frequency in AIH-1 patients compared to controls. This finding, in combination with data from the literature on other autoimmune disorders, provides support for the direct involvement of KIR2DS1-positive NK cell clones in the pathogenesis and progression of AIH-1.

The activation of NK cells expressing the cell surface receptor KIR2DS1 is strictly dependent upon the interaction of this receptor with its ligands, particularly HLA-C2.

Previous studies suggest that the HLA-C2 high affinity ligands for KIR2DS1 contribute to the development of specific autoimmune disorders such as ankylosing spondylitis [53] and psoriasis vulgaris [54].

In our AIH-1 patients we found a high frequency of the KIR-ligand combination KIR2DS1/HLA-C2 present.

Therefore, it is likely that HLA-C2 molecules also have a role in AIH-1, not only by promoting the presentation of hepatic antigens to CD4^+^ and CD8^+^ T Cells but also through interaction with their specific KIR receptors.

Another interesting finding emerging from our study is the reduced presence of two combinations of inhibitory KIR genes and their ligands (*KIR2DL3*/HLA-C1 and *KIR3DL1*/HLA-Bw4). Within such an immunogenetic framework, activating signals may tip the balance and override inhibitory signals, thereby promoting undesired NK cell autoreactivity.

This hypothesis is supported by several findings in the literature. In seronegative individuals exposed to HIV infection it would seem that the reduced frequency of *KIR3DL1* and its HLA-Bw4 ligand offers protection against this viral infection by increasing NK cell activation [55].

A decreased frequency of the KIR-ligand combination KIR3DL1/HLA-Bw4 has also been found in patients with PSC [56] which furthermore confirms that KIRs and their HLA ligands are likely to have a contributory role in the immunological pathways underlying the development and progression of autoimmune liver disease.

In our study the KIR gene *KIR2DS1* was found associated with the development of AIH-1, particularly with early onset of the disease (Fig 1). Interestingly and albeit not reaching statistically significance, patients homozygous for the HLA-C2 ligand group (KIR2DS1/HLA-C2/C2) were more likely to be diagnosed at a later age than patients pertaining to the other ligand groups (KIR2DS1/HLA-C1/C1 or HLA-C1/C2). This may possibly be explained by lower NK cell cytotoxicity in the presence of this KIR-ligand combination as shown in a study of 1277 patients treated with allogeneic hematopoetic stem cell transplantation for myeloid leukemia. These authors found that reduced NK cell alloreactivity related to this immunogenetic profile (KIR2DS1/HLA-C2/C2) increased the risk of relapse [57].

In peripheral blood, NK cells constitute up to about 10% of the lymphocyte population but in liver tissue this percentage rises to over 30% since these cells have a fundamental role in protecting liver parenchyma against injury deriving from the direct or indirect attack of viruses and are involved in maintenance of immune tolerance towards numerous gut-derived foreign antigens [58].

There is a growing amount of evidence demonstrating how innate immunity and NK cells are implicated in the pathogenesis of PBC [59, 60].

However, little is known about the role of NK cells in AIH. Most researchers have focused their attention on adaptive immune response mechanisms and the damage to tissue induced by CD4^+^ and CD8^+^ T cells. However, it has been shown that NK cells can directly attack liver parenchyma or contribute to damage through cytokine secretion and/or cell to cell contact [61].

In our study, the reduced frequency of HLA-Bw4 ligands for KIR3DL1, HLA-C1 ligands for KIR2DL3 and the high frequency of the HLA-C2 high affinity ligands for KIR2DS1 probably contributes to the development and progression of AIH-1 by creating an imbalance in favour of activating NK-cell receptor signaling. The important predictive potential observed for the *KIR2DS1* activating KIR gene makes it suitable for use as a genetic marker of early onset AIH-1.

Taken together, our data provide several new insights into the immunogenetic features of AIH-1 and may encourage researchers to setup innovative in vitro studies as well as immunohistochemical experiments to better understand how KIR receptors alter NK cell function in autoimmune liver disease. However, it needs to be considered that KIR receptors, although prevalently expressed by NK cells, can also be expressed by a distinct lineage of CD8^+^ T cells [62]. Therefore, it cannot be excluded that also these cells have a role in the complex mechanisms leading to AIH-1.
